# Is using the Gail model to calculate the risk of breast cancer in the Venezuelan population justified?

**DOI:** 10.3332/ecancer.2023.1590

**Published:** 2023-08-21

**Authors:** Josepmilly del Valle Peña Colmenares, Carmen Cristina García, Yazmin José Velásquez Velásquez, Leider Arelis Campos Pino, Álvaro Gómez Rodríguez, Wladimir José Villegas Rodríguez, David José González Vargas, Douglas José Angulo Herrera

**Affiliations:** 1 Servicio Patología Mamaria del Servicio Oncológico Hospitalario (SOH), Instituto Venezolano del Seguro Social (IVSS), Caracas 1040, Venezuela; 2 Cátedra de Patología General y Fisiopatología, Escuela Luis Razetti, Facultad de Medicina, Caracas 1050, Venezuela; 3 Servicio Oncológico Hospitalario (SOH), Instituto Venezolano del Seguro Social (IVSS), Caracas 1040, Venezuela; 4 Escuela de Estadística y Ciencias Actuariales, Universidad Central de Venezuela, Caracas 1050, Venezuela; a https://orcid.org/0000-0002-1114-6289; b https://orcid.org/0000-0002-7889-9445; c https://orcid.org/0000-0003-3307-2564; d https://orcid.org/0000-0002-0907-8467; e https://orcid.org/0000-0003-3740-0238; f https://orcid.org/0000-0001-8999-9751; g https://orcid.org/0000-0001-8071-3139; h https://orcid.org/0009-0003-5506-0297

**Keywords:** Gail model, breast cancer, young women, risk factor

## Abstract

**Objective:**

To evaluate the accuracy of the Gail model (GM) in women who already have a diagnosis of breast cancer (BC) from the Breast Pathology Service, Hospital Oncology Department of the Venezuelan Social Security Institute (SOH-IVSS) in the period 2004–2014. To compare the accuracy of the GM in women aged above and below 40 years with a diagnosis of BC.

**Method:**

Descriptive, retrospective, cross-sectional, 830 records of patients diagnosed with BC were reviewed between 2004 and 2014.

**Results:**

The mean age for diagnosis of the disease was 46 ± 13 years; menarche age was 13 years ± 2; age at first birth 22 ± 5 years, with a history of biopsy 32 ± 11, the percentage of relatives with a primary history of BC reported (PHBC) 9.3%. Only 41% of women with a diagnosis of BC reported Gail >1.67 (positive Gail). In the dichotomous logistic regression that related positive Gail with the independent variables, it was observed: greater probability of positive Gail if menarche age <11 years (*p* < 0.036), PHBC (*p *= 0.005), previous biopsy (*p* = 0.007), age at first birth 25–29 years (*p *= 0.019). When stratifying by age, unlike the bivariate analysis, women over 40 years of age are more likely to have a positive Gail in menarche age <11 years (*p *= 0.008), PHBC (*p *= 0.001), previous biopsy (*p *= 0.025) when compared with younger women, the age at first birth between 25 and 29 years was statistically significant for both groups; however, the probability was higher in younger women (*p *= 0.008).

**Conclusion:**

There is no conclusive evidence to consider that the GM is applicable to Venezuelan women due to its low precision since it only identified 41% of the patients who had BC as high risk; however, when the factors are analysed separately, we found a higher probability of a positive Gail with statistical significance in EM <11 years, PHBC, previous biopsy and age at first birth 25–29 years; When stratifying by age, we observed that the age at first birth 25–29 years in women aged 40 or less increases the probability of a positive Gail. It is necessary to develop new risk assessment models that are adapted to our female population.

## Introduction

Breast cancer (BC) is the most common cancer in women, with high morbidity and mortality rates [1]. In 2020, Globocan* figures showed that BC accounted for 15.5% of cancer incidence and 10.5% of cancer deaths in Venezuela. The standardised incidence by age was 52.6 per 100,000 residents [2]. For over three decades, reports have described risk assessment models that make it possible to estimate the absolute risk an individual will develop BC and identify women at high risk (HR). That data make it possible to design prevention trials in HR population subgroups that can help identify detection and prevention strategies [3, 4].

Quantitative risk estimation is possible because multiple risk factors (RFs) can be combined into a comprehensible risk expression. The Gail and Claus models are the most widely used tools for estimating quantitative risk [5]. Gail *et al* [3, 6, 7] described a risk-assessment model that focuses mainly on non-genetic RFs, with limited information on family history. The Gail model (GM) was first designed to determine eligibility for a BC prevention trial and was one of the first models for estimating BC risk, but reports have described its limitations. The GM has since been modified and validated in different settings [3, 6, 8–9].

Current models can give a fairly accurate estimate of the risk of developing BC and can inform women with sufficient certainty if they have an increased lifetime risk. But it remains impossible to determine which patients will develop cancer and which patients will not [5]. Risk-estimation algorithms therefore now include more parameters, such as mammographic density [10], various single nucleotide polymorphisms [11] and other lifestyle-related factors to improve their accuracy. But more studies with large patient cohorts are vital for validating these new criteria.

We must remember that all risk-estimation tools have limitations, and while various BC risk models have emerged in the last 20 years, their use is not common in Venezuelan clinical practice. Nonetheless, we can establish appropriate guidelines for applying these models as their use grows more common. Our study applied the GM to a group of patients already diagnosed with BC, allowing us to evaluate the accuracy of the GM and if applying it to the Venezuelan population is feasible.

### General objectives

1. To evaluate the accuracy of the GM in women with a BC diagnosis from the Breast Pathology Service, Hospital Oncology Department, Venezuelan Institute of Social Security (SOH-IVSS).
2. To compare the accuracy of the GM in women over and under age 40 with a BC diagnosis.
3. To analyse if the variables that the GM include – percentage of relatives with a primary history of BC reported (PHBC), age at menarche (AM), age at first delivery (AFD) and previous biopsy (PB) – were associated with a higher BC risk.

## Method

This is a descriptive, retrospective, cross-sectional study of 830 patients with a BC diagnosis at the Breast Pathology Service, SOH-IVSS, covering two periods and including two patient groups. From 2004 to 2014, the study included 415 women aged 40 and below, and from 2008 to 2012, the study included 415 women aged 41 and over. Risk estimates were calculated using the computerised GM [12]. A Gail risk >1.66% was considered high and designated Gail positive, while a Gail risk of ≤1.66 Gail was designated Gail negative. BC risks of all patients were calculated separately using the GM.

### Statistical analysis

Means and SD were calculated for continuous variables and frequencies and percentages were calculated for nominal variables. The McNemar test was applied to verify differences in the GM between white and black patients. The estimate of Gail-positive probability was determined using a dichotomous logistic regression model, and the validity of that model was verified using the Hosmer-Lemeshow test. Confidence intervals were adjusted to infinity using the Rindskopf procedure. A value of *p* < 0.05 was considered statistically significant. Study data were tabulated and processed using SPSS 25.

## Results

Eight hundred and thirty patients met the inclusion criteria in the Breast Pathology Service, SOH-IVSS from 2004 to 2014. The average age at BC diagnosis was 46 ± 13 years. The average ages for GM variables were AM 13 ± 2 years, AFD 22 ± 5 years and PB 32 ± 11 years; 23% of patients (191) had relatives with a history of breast cancer (HBC); 40.3% of those relatives were first degree; and 26.5% had a history of other cancers. Notably, only 41% of the women with BC diagnosis had a Gail score >1.66. Of all patients, 92.2% with a PHBC, 72.2% with a PB, 54% with AM <11 years and 82.11% with AFD from 25 to 29 years were Gail positive.

### Relationship between the GM and the different variables

A comparison of patients' ages ≤40 with patients’ ages ≥41 found no difference between a positive or negative Gail score, but four other GM variables – AM, PHBC, PB and AFD – were all statistically significant. No difference was found when including patients who had relatives with another type of cancer (Table 1).

### Bivariate analysis: Gail and odds ratio

Performing a bivariate analysis and relating the independent variables (AM, PHBC, PB, AFD and others) with the GM revealed a higher probability of being Gail positive with a lower AM (<11 years OR: 2.01/*p* < 0.001), PHBC (OR: 5.85/*p* < 0.01), PB (OR: 1.79/*p* < 0.006) and AFD >25 years (25–29 years OR: 4.48/*p* < 0.007; ≥ 30 years OR: 7.87/*p* < 0,001), with all variables statistically significant. But for patients with AM ≥14 years (OR: 0.22/*p* < 0.001), no PB (OR: 0.47/*p* < 0.039) and AFD ≤24 years (OR: 0.21/*p* < 0.008), the probability of being Gail positive decreased 0.22 times, 0.47 times and 0.21 times, respectively. The bivariate analysis found no statistical association between being Gail positive and age (≤ or >40 years). This analysis included other variables, such as second-degree relatives with BC and other cancers, for which no relationship was found (Table 2).

### Bivariate analysis: GM > and <40 years with odds ratio (Table 3)

Notably, stratifying patients by age with a cutoff at age 40 showed that women under 40 were more likely to be Gail positive (OR: 2.48/*p* = 0.001) with an AM ≤11 years. In contrast, there was no statistical association between age and AM amongst women aged ≥41 years (OR: 1.55/ *p* = 0.072).

Also notably, younger women (≤40) with a PHBC (OR: 7.47/*p* = 0.002) had an 88% probability of being Gail positive compared to older women (OR: 2.89/*p* = 0.002), which was statistically significant. Similarly, women aged £40 years with a PB for any disease were 2.32 times more likely to be Gail positive (OR:2.32/*p* = 0.048).

This analysis also included AFD, another GM variable, and found that women >40 years with an AFD >30 years were 7.14 times more likely to be Gail positive (*p* = 0.001). Women aged ≤40 years were 5.87 times more likely to be Gail positive (*p* = 0.013). The bivariate analysis also found that while the probability of being Gail positive fell with a lower AFD, the difference was greater in women aged>40 years, with a statistical association (AFD 20–24 OR: 0.33/*p* = 0.002).

### Dichotomous logistic regression relating the GM with independent variables

The multivariable regression analysis found that women with AM <11 years, relatives with a family HBC, PHBC, PB and AFD 25–29 years were 3.98 (*p* = 0.036), 2.99 (*p* = 0.014); 4.06 (*p* = 0.005), 3.09 (*p* = 0.007) and 4.05 (*p* = 0.019) times more likely to be Gail positive than women with AM >11 years with no family HBC or PB or whose AFD <25 years. All these findings were statistically significant (Table 4).

### Dichotomous logistic regression relating the GM to age ( > and ≤ 40 years)

Using dichotomous regression analysis to compare the GM to the different variables by age (> or ≤40 years) again found that women aged ≥41 years with AM ≤11 years OR:5.05/*p* = 0.008), PHBC (OR:7.05/*p* = 0.001), PB (OR:4.67/*p* = 0.025) were more likely to be Gail positive than younger women. The difference between the two groups was statistically significant for all variables, though PHBC in women under the age of 40 years was less significant. However, younger women were significantly more likely to be Gail positive, despite having an AFD from 25 to 29 years (Table 5).

Notably, only 41% of our study population was classified as HR. Evaluating all the factors used in this calculation, we note that both the bivariate analysis and the multivariable regression model found significant differences in all factors the GM includes. Unlike the bivariate analysis, the dichotomous logistic regression model found, when stratifying by age, that women aged >40 years were more likely to be Gail positive and had a lower AM, a PHBC and a PB when compared with younger women. AFD from 25 to 29 years was statistically significant for both groups.

## Discussion

The accuracy of a risk model depends on identifying RFs, estimating their effects in specific populations and knowing the medical, family and demographic history of women [13]. However, the socioeconomic situation in Venezuela and Latin America is also important when considering the use of risk models. The regional context justifies using methods considered outdated in developed countries, where advanced technologies such as artificial intelligence are more common and make the GM useful in studying populations such as Venezuelan women.

Notably, the GM statistical algorithm was developed after a screening study of 280,000 women ages 35–74 and has since undergone multiple modifications. Researchers have also noted that the GM does not consider racial or ethnic differences, BRCA genetic variants, tamoxifen use, or women excluded from the GM who already had a confirmed diagnosis of ductal or lobular carcinoma of the breast *in situ*. Nonetheless, research has shown that the GM is a reasonable tool for estimating BC risk in white women, and other researchers have enhanced the GM to provide accurate risk assessments for African-American, Hispanic and Asian women. The Venezuelan population is characterised by a mix of three ethnic groups (white, Indian and black) for which the GM has not been validated [5, 13–15].

BC is a complex disease resulting from the interaction of multiple environmental, hormonal and lifestyle factors, plus the genome of each patient. Clinicians and researchers consider a broad range of RFs but cannot determine the causative agent in 75% of BC cases [16]. Carcinogenesis results from a complex combination of genome and host factors and the response of the body to DNA and to cell damage [16–19].

There are reports that years of exposure to oestrogens and the increase in ovulatory cycles associated with early AM is an RF for BC [3, 14, 17, 18]. Pregnancy at an early age has also been defined as an RF, and pregnancy before age 20 is associated with a markedly reduced BC risk [13, 20]. Some researchers have suggested that this ‘protector’ effect is found only when pregnancies end in the birth of a viable foetus [5]. In contrast, nulliparity and AFD >30 years are associated with an increased risk of subsequent BC [5, 20].

BC has always been considered a multifactorial disease. However, having relatives with an HBC is another variable that has been studied, and has been called a non-modifiable RF. The risk of developing BC is strongly associated with the number and type of relatives affected, and their age at diagnosis [5, 13, 20, 21]. In this study, 23% of patients had relatives with an HBC, and 40.3% of them were first-degree relatives. Notably, just 9.2% of study patients had a PHBC, but 92.2% of those patients were Gail positive.

To date, few studies have evaluated risk-estimation tools in patients who already have a BC diagnosis. Pastor *et al* [15] used the National Surgical Adjuvant Breast and Bowel Project (NSABP) model, an adaptation of the GM, to estimate the 5-year risk of developing BC in 186 incident BC cases. That study found the NSABP would only have identified 40% of women with a BC diagnosis as HR. Ateş *et al* [22] reported similar results, which showed the risk analysis method they studied did not detect 67.48% of BC patients. That finding was similar to our study, which reported only 41% of patients as Gail positive. The GM would thus not have identified 59% of our patients as HR before their diagnosis, compared to the average risk of the population of the same age.

### Comparison of the GM in women > or <40 years of age

To date, few studies have evaluated the GM as a population screening tool. Nickson *et al* [23] used data from 40,158 women aged 50–69 years who participated in the BreastScreen Australia Programme. The authors examined the association between Gail scores and future invasive BC, comparing the observed and expected results for groups classified by Gail score. That study confirmed that the GM can effectively stratify a population aged 50–69 by the risk of future invasive BC.

Several methods for determining BC risk have been identified, and the two most common are the GM, including modified versions, and the National Cancer Institute (NCI) method. Studies have found these models valid in many European racial and ethnic groups, but some of those studies did not include women under age 35, and neither does the NCI model [8, 12, 13].

Mackarem *et al* [24] evaluated 124 patients under the age of 40 years treated for carcinoma in situ and invasive cancer from 1983 to 1995. This study also compared this group with two other cohorts of women under age 40 years and verified that the GM provided little useful information in this age group. Similar to our study, Mackarem *et al* [24] found the relative risk of developing BC rose both as AM fell and in women with a PB. However, BC risk level depends on whether the patient is over or under the age of 50 years. Our bivariate analysis found that patients under the age of 40 years with an AM <11 years, a PHBC and a PB were more likely to be Gail positive than women over the age of 40 years. AM, PHBC and PB were all statistically significant in both groups, but the dichotomous logistic regression model found that AFD from 25 to 29 years (OR: 9.57/*p* = 0.008) in women under the age of 40 years increased the probability of being Gail positive. Notably, we found no significant difference between the two study groups when we used age as the cohort cutoff point.

The GM was designed using a population of cases and controls from the breast cancer detection project (BCDP). Women under the age of 40 years were only 3.8% of the BCDP population with BC. However, BC frequency in young women in developed countries varies from 5% to 6%, while in developing countries, it varies from 18% to 20%. This difference could explain why the GM has not been validated in women under the age of 40 years [3, 24–31].

It seems logical that all risk models apply to women with higher BC risk, and to various research programmes. Projects or studies in populations of women under the age of 40 years, in which BC percentage is under 20% [26, 30–34], would thus lead us to ask ‘Will this study also fail to validate the GM in this patient subgroup? Will BC detection and prevention continue to exclude young women?’

### Risk assessment

Assessing BC risk requires a deep knowledge of RFs, family tree analysis, comparative statistics, genetic susceptibility testing and probability science [5].

Calculations of absolute individual BC risk are not common but providing appropriate clinical management for women requires knowing that risk [35]. BC has been the leading cause of cancer morbidity and mortality in Venezuelan women since 2009 [32]. Venezuela lacks updated registries and prevention policies and thus needs risk-estimation methods that allow us to predict the probability a woman will develop BC relative to the general population.

All patients in our study already had a BC diagnosis, and the GM could thus not identify 59% as HR. We could thus conclude that using the GM in Venezuelan women seems inappropriate.

Risk assessment and risk management involve scientific and public beliefs and issues of power and trust. Policymakers who want to promote educational and interventional strategies to reduce health risks must understand the different ways the public and health professionals perceive risks [13, 36].

### Usefulness of GM in the era of artificial intelligence

In the last two decades, BC research has focused on early detection methods and sought treatments, achieving significant progress. However, BC remains the leading cause of morbidity and mortality in women in Venezuela and globally [5, 15]. One of the newest scientific and technological advances is the global development of various artificial intelligence tools for diagnosis (screening, artificial intelligence based on computer-aided diagnosis, imaging control and dose reduction, radiological-pathological correlations, etc.) and establishing the probability of determining the risk a woman will develop BC [37, 38]. The validity of using these tools either individually or with traditional risk-assessment methods is the subject of debate. Venezuela has suffered a steady decline in its economy and basic social services amid a humanitarian crisis worsened by the COVID-19 pandemic. The country thus lacks public health policies and its public health facilities either lack new technologies for their specialist physicians to use, or those technologies arrive late. Consequently, the benefits of technology do not reach most Venezuelans, who rely on public health facilities for care.

Venezuela also lacks a working BC screening programme. We know that risk-stratified BC detection protocols require accurate risk estimates that draw on data that is easily accessible from population-based programmes [39]. Venezuela lacks clear policies for monitoring and caring for BC patients, and such policies require guidelines for BC screening [16].

It is possible to obtain the data the GM requires, so implementing the GM broadly in many healthcare centres that serve low-income patients remains vital. And notably, other BC risk-estimation models are based on the GM, specifically the breast cancer risk assessment tool, which is available in the website of the NCI of the U.S. National Institutes of Health.

It is therefore critical to create a new BC prevention strategy. Our study opens a line of research in Venezuela that relies on using simple predictive methods for obtaining data, variables, or other factors the GM incorporates that could be useful in developing new risk algorithms. This line of research could prevent Venezuela from having to adapt guidelines for Venezuelan women and instead lead to similar studies in public and private healthcare facilities. That research could examine other independent variables and help create a new risk model appropriate for Venezuelan women.

## Observations

Our study has several limitations: a) It was a retrospective study that collected data from patients diagnosed with BC, not a prospective study conducted in a population screening context. This study did not include healthy women with follow-up to assess observed/expected cases. b) We know of other models or new BC risk calculation algorithms, specifically the Tyrer Cuzick Calculator, but we used the GM because it was based on the only data obtained from histories of breast disease at the hospital where we conducted the study. That hospital lacks genetic counselling, a geneticist, and genetic testing, which were thus unavailable for our study. We also could not consider mammographic density because patients had mammograms in different health centres. These and other limitations forced us to use one of the earliest GM methods, which some doctors might consider obsolete compared to more recent technologies. c) Some authors caution that a cutoff point at age 40 in the GM is not useful and recommend against it. Our study used the age of 40 years as a cutoff point and had the same number of patients in the two age groups, as we tried to stratify our study population by age to determine any differences. Risk models usually exclude women under the age of 40 years due to the low incidence of BC in that subgroup. d) Our study data are not generalisable as the study sample is from a single hospital, though that hospital is a national breast carcinoma referral centre.

## Conclusion

Risk models are used to help advise women about their risk or to identify patients who need a referral to genetic or HR specialists. Venezuela lacks studies that allow us to evaluate the various predictive BC risk models. It is thus useful to apply the GM in our population, as risk-estimation tools are important for decision-making in BC prevention and detection.

The GM only identified 41% of patients with BC as HR. Given such low accuracy, the GM does not seem appropriate for Venezuelan women. However, by analysing RFs separately, we found a greater probability of a positive Gail score, with statistical significance in AM <11 years, PHBC, PB and AFD 25–29 years.

We know that currently used risk models have been developed in other latitudes and were validated in populations that do not necessarily have the same characteristics as ours. Developing a new risk-assessment model is thus essential. That model should include the aforementioned variables, add other RFs that the GM risk calculation does not include and contain enough cases to allow us to set appropriate guidelines for Venezuelan women.

## Conflicts of interest

The authors declare that there are no conflicts of interest.

## Disclosure

The authors have no financial interest to declare related to the disclosure of the content of this article. Their only interest focuses on opening a line of research on the topic of the study in Venezuela.

## Funding

No financial support was received for writing this article, for data collection or analysis, or for preparing or approving this current manuscript.

## Figures and Tables

**Table 1.** Relationship between the GM and the different variables.

		GAIL positive		GAIL positive		
Variables	Answers	*n*	%	*n*	%	*p*
Age	>40 years	159	46.8	256	52.2	0.120
	≤40 years	181	53.2	234	47.8	
AM	≤11 years	107	31.5	91	18.6	<0.001
	12–13 years	198	58.2	229	46.7	
	≥14	35	10.3	170	34.7	
HBC	Yes	112	32.9	79	16.1	<0.001
	No	228	67.1	411	83.9	
Relatives	First degree	72	64.3	5	6.3	<0.001
	Second degree	40	35.7	74	93.7	
PB	Yes	13	3.8	5	1.0	<0.001
	No	327	96.2	485	99.0	
Age of first childbirth	<20 years	20	5.9	220	44.9	<0.001
	20–24 years	36	10.6	215	43.9	
	25–29 years	124	36.5	27	5.5	
	≥30 years	53	15.6	1	0.2	
Other types of cancer in the family	Yes	87	25.6	131	26.7	0.712
	No	253	74.4	359	73.3	

**Table 2.** Gail and odds ratio (bivariate analysis).

Variables	Answers	OR	IC – 95%		*p*
Age	>40 years	0.80	0.61	1.06	0.120
	≤40 years	1.25	0.94	1.64	
AM	≤11 years	2.01	1.46	2.78	<0.001
	12–13 years	1.59	1.20	2.10	<0.001
	≥14	0.22	0.15	0.32	<0.001
HBC	Yes	1.64	1.40	1.93	<0.001
	No	0.64	0.54	0.77	<0.001
Relatives	First degree	5.85	1.39	12.57	<0.001
	Second degree	0.75	0.50	1.13	0.170
PB	Yes	1.79	1.33	2.42	0.006
	No	0.47	0.22	0.98	0.039
Age of first childbirth	<20 years	0.10	0.06	0.17	0.002
	20–24 years	0.21	0.14	0.31	0.008
	25–29 years	4.48	1.24	9.57	0.007
	≥30 years	7.87	2.07	13.89	0.001
Other types of cancer in the family	Yes	0.78	0.49	1.87	0.287
	No	0.33	0.11	0.69	0.845

**Table 3.** GM > and <40 years with odds ratio (bivariate analysis).

Variables	Answer	OR	IC – 95%		*p*
AM >40	≤11 years	1.55	0.96	2.49	0.072
	12–13 years	1.60	1.07	2.38	0.021
	≥14 years	0.37	0.23	0.60	<0.001
AM <40	≤11 years	2.48	1.59	3.88	<0.001
	12–13 years	1.56	1.05	2.31	0.002
	≥14 years	0.08	0.03	0.19	<0.001
HBC >40	Yes	2.34	1.88	2.90	<0.001
	No	0.44	0.32	0.60	0.002
HBC <40	Yes	1.17	0.92	1.48	0.213
	No	0.88	0.71	1.09	0.345
Relatives >40	First degree	2.89	2.42	3.43	0.002
	Second degree	0.93	0.47	1.86	0.845
Relatives <40	First degree	7.47	2.13	16.87	0.002
	Second degree	0.62	0.37	1.04	0.067
PB >40	Yes	1.79	1.22	2.62	0.021
	No	0.53	0.26	1.09	0.038
PB <40	Yes	2.32	2.07	2.59	0.048
	No	0.29	0.13	1.07	0.386
Age of first childbirth >40	<20	0.13	0.07	0.25	<0.001
	20–24	0.33	0.20	0.56	0.002
	25–29	5.47	1.55	9.59	0.002
	≥30	7.14	2.14	11.23	<0.001
Age of first childbirth <40	<20	0.07	0.03	0.17	<0.001
	20–24	0.12	0.06	0.23	0.002
	25–29	3.42	1.32	7.69	0.033
	≥30	5.87	1.23	8.25	0.013
Other family member with cancer >40	Yes	0.42	0.13	0.99	0.174
	No	0.11	0.03	0.33	0.315
Other family member with cancer <40	Yes	0.79	0.55	1.37	0.339
	No	1.11	0.97	1.87	0.287

**Table 4.** Dichotomous logistic regression relating the GM with independent variables.

Variables	OR	IC – 95%		*p*
AM ≤11 years	3.98	1.02	9.05	0.036
HBC	2.99	1.07	10.08	0.014
First-degree relative	4.06	1.37	11.09	0.005
PB available	3.09	1.69	9.17	0.007
Age at first childbirth: 25–29 years	4.05	1.19	11.25	0.019

**Table 5.** Dichotomous logistic regression relating Gail positive result age.

	>40 years				≤40 years			
Variables	OR	IC – 95%		*p*	OR	IC – 95%		*p*
AM ≤11 years	5.05	1.99	12.05	0.008	2.05	1.08	7.88	0.036
HBC	3.25	1.16	9.23	0.004	1.07	0.99	4.85	ns
First-degree relative	7.05	2.04	17.45	< 0,001	4.08	2.01	13.52	0.005
PB available	4.67	1.87	9.07	0.025	1.77	0.98	3.57	ns
Age at first childbirth: 25–29 years	5.04	2.04	13.25	0.002	9.57	1.28	13.25	0.008

## Footnotes

* The only statistical BC data 2020 are published by Globocan. The Ministry of Popular Power for Health (MPPS) is the only body legally authorised to publish the national epidemiological bulletin but has not done so since 2014. This study thus does not include updated BC morbidity and mortality data from more recent years.

## References

[1] GLOBOCAN: estimated cancer incidence, mortality and prevalence worldwide in 2012. http://globocan.iarc.fr/Pages/summary_table_pop_sel.Aspx.

[2] Venezuela, BOLIVARIAN REPUBLIC OF – International Agency for Research on Cancer.

[3] Gail MH, Brinton LA, Byar DP (1989). Projecting individualized probabilities of developing breast cancer for white females who are being examined annually. J Natl Cancer Inst.

[4] Wang X, Huang Y, Li L (2018). Assessment of performance of the Gail model for predicting breast cancer risk: a systematic review and meta-analysis with trial sequential analysis. Breast Cancer Res.

[5] Sakorafas GH, Krespis E, Pavlakis G (2002). Risk estimation for breast cancer development; a clinical perspective. Surg Oncol.

[6] Costantino JP, Gail MH, Pee D (1999). Validation studies for models projecting the risk of invasive and total breast cancer incidence. J Natl Cancer Inst.

[7] Evans G, Brentnall A, Harvi M (2014). Breast cancer risk in young women in the national breast screening programme: implications for applying NICE guidelines for additional screening and chemoprevention. Cancer Prev Res.

[8] Euhus DM, Leitch AM, Huth JF (2002). Limitations of the Gail model in the specialized breast cancer risk assessment clinic. Breast.

[9] Claus EB, Risch N, Thompson WD (1993). The calculation of breast cancer risk for women with a first-degree family history of ovarian cancer. Breast Cancer Res Treat.

[10] Judkins T, Rosenthal E, Arnell C (2012). Clinical significance of large rearrangements in BRCA1 and BRCA2. Cancer.

[11] Kast K, Schmutzler RK, Rhiem K (2014). Validation of the manchester scoring system for predicting BRCA1/2 mutations in 9,390 families suspected of having hereditary breast and ovarian cancer. Int J Cancer.

[12] Detailed breast cancer risk calculator. https://www.merckmanuals.com/medical-calculators/Gail99-es.htm.

[13] Seyednoori T, Pakseresht S, Roushan Z (2012). Risk of developing breast cancer by utilizing Gail model. Women & Health.

[14] McTiernan A, Kuniyuki A, Yasui Y (2001). Comparisons of two breast cancer risk estimates in women with a family history of breast cancer. Cancer Epidemiol Biomarkers Prev.

[15] Pastor I, Morales M, Llopis A (2005). Aplicación del método de Gail de cálculo de riesgo de cáncer de mama a la población valenciana. Clin Transl Oncol.

[16] Peña CJ, Pérez J, Jahon J (2017). Consenso de detección Temprana de Cáncer de Mama. Pesquisa oportunista. Pesquisa en Cáncer de mama. Rev Venez Oncol.

[17] McTiernan A (2003). Behavioral risk factors in breast cancer: can risk be modified?. Oncologist.

[18] Prentice RL, Caan B, Chlebowski RT (2006). Low-fat dietary pattern and risk of invasive breast cancer: the women’s health initiative randomized controlled dietary modication trial. JAMA.

[19] Pruthi S, Brandt K, Degnim A (2007). A multidisciplinary approach to the management of breast cancer, part 1: prevention and diagnosis. Mayo Clin Proc.

[20] Vogel VG (2000). Breast cancer prevention: a review of current evidence. CA Cancer J Clin.

[21] Azzena A, Zen T, Ferrar A (1994). Risk factors for breast cancer. Case-control. Study results. Eur J Gynaecol Oncol.

[22] Ateş E, Bozkurt B, C7;am R (2018). Sensitivities of the Gail, NSABP and NCI risk analysis models for Turkish women for breast cancer risk assessment. Ankara Med J.

[23] Nickson C, Procopio P, Velentzis L (2018). Prospective validation of the NCI breast cancer risk assessment tool (Gail Model) on 40,000 Australian women. Breast Cancer Res.

[24] MacKarem G, Roche CA, Hughes KS (2001). The effectiveness of the Gail model in estimating risk for development of breast cancer in women under 40 years of age. Breast J.

[25] Baker LH (1982). Breast cancer detection demonstration project: five-year summary report. CA Cancer J Clin.

[26] Narod SA (2012). Breast cancer in young women. Nat Rev Clin Oncol.

[27] Anders CK, Fan C, Parker JS (2011). Breast carcinomas arising at a young age: unique biology or a surrogate for aggressive intrinsic subtypes?. J Clin Oncol.

[28] Paluch-Shimon S, Pagani O, Partridge AH (2017). ESO-ESMO 3rd international consensus guidelines for breast cancer in young women (BCY3). Breast.

[29] Partridge AH, Pagani O, Abulkhair O (2014). First international consensus guidelines for breast cancer in young women (BCY1). Breast.

[30] Peres J (2013). Advanced breast cancer in young women. J Ntl Cancer Inst.

[31] Franzoi M, Rosa D, Zaffaroni F (2019). Advanced stage at diagnosis and worse clinicopathologic features in young women with breast cancer in Brazil: a subanalysis of the AMAZONA III study (GBECAM 0115). J Glob Oncol.

[32] Capote Negrín LG (2015). Resumen del cáncer en Venezuela 2012. Rev Venez Oncol.

[33] Cazap E (2018). Breast cancer in Latin America: a map of the disease in the region. Am Soc Clin Oncol Educ Book.

[34] Villarreal-Garza C, Mohar A, Bargallo-Rocha J (2017). Molecular subtypes and prognosis in young Mexican women with breast cancer. Clin Breast Cancer.

[35] Tyrer J, Duffy S, Cuzick J (2004). A breast cancer prediction model incor- porating familial and personal risk factors. Stat Med.

[36] Katapodi MC, Lee KA, Facione NC (2004). Predictors of perceived breast cancer risk and the relation between perceived risk and breast cancer screening: a meta-analytic review. Prev Med.

[37] Morgan MB, Mates JL (2021). Applications of artificial intelligence in breast imaging. Radiol Clin North Am.

[38] Fernandez De Freitas M, Capecchi A (2021). Inteligencia artificial en la detección del cáncer de mama por tomosíntesis, ¿hacia dónde vamos?. Rev Cient CMDLT.

[39] Australian Institute of Health and Welfare (2017). Breast Screen Australia Monitoring Report 2014–2015. Cancer Series No. 106. Cat. No. CAN 105.

